# Improving water dispersibility and bioavailability of luteolin using microemulsion system

**DOI:** 10.1038/s41598-022-16220-4

**Published:** 2022-07-13

**Authors:** Ayaka Miyashita, Junya Ito, Isabella Supardi Parida, Naoki Syoji, Tomoyuki Fujii, Hidehiro Takahashi, Kiyotaka Nakagawa

**Affiliations:** 1 Laboratory of Food Function Analysis, Graduate School of Agricultural Science, Tohoku University, 468-1 Aramaki Aza Aoba, Aoba-ku, Sendai, Miyagi 980-8572 Japan; 2 Center for the Cooperation of Community Development and Research Promotion, Miyagi University, Sendai, Miyagi 981-3298 Japan; 3 Laboratory of Terahertz Optical and Food Engineering, Graduate School of Agricultural Scierence, Tohoku University, Sendai, Miyagi 980-8572 Japan; 4 PetroEuroAsia, Suntou, Shizuoka 441-0907 Japan

**Keywords:** Nutrition, Drug delivery

## Abstract

We have studied the physiological effects and health functions of luteolin, especially focusing on its absorption and metabolism. Recent studies have reported the advantages of microemulsion to improve the bioavailability of poorly water-soluble compounds, including luteolin. In the present study, we aimed to evaluate the absorption and metabolic profile of luteolin delivered in microemulsion system via oral intake. First, we prepared water-dispersed luteolin (WD-L) using a microemulsion-based delivery system and confirmed that WD-L has superior water dispersibility compared to free luteolin (CO-L) based on their particle size distributions. Following administration of WD-L and CO-L to rats, we detected high level of luteolin-3'-O-β-glucuronide and lower levels of luteolin, luteolin-4'-O-β-glucuronide, and luteolin-7-O-β-glucuronide in plasma from both CO-L and WD-L groups, indicating that the metabolic profile of luteolin was similar for both groups. On top of that, we found a 2.2-fold increase in the plasma area under the curve (AUC) of luteolin-3'-O-β-glucuronide (main luteolin metabolite) in WD-L group (vs. CO-L). Altogether, our results suggested that delivering luteolin by microemulsion system improve its oral bioavailability without affecting its metabolite profile. This evidence thereby provides a solid basis for future application of microemulsion system for optimal delivery of luteolin.

## Introduction

**Figure 1:**
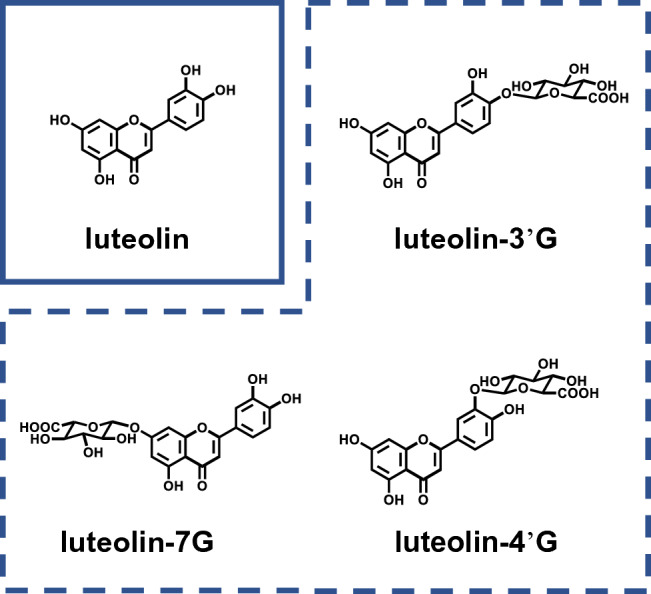
Chemical structures of luteolin, luteolin-3'G, luteolin-4'G, and luteolin-7G.

Luteolin (3',4',5,7-tetrahydroxyflavone, Fig. 1) is a flavonoid that ubiquitously presents as glucosides in bell pepper, perilla, and celery^1–3^. Various studies have reported the anti-inflammatory, antioxidative, and anticancer activities of luteolin, making it a viable therapeutic option to improve human health^4–8^. To comprehend how luteolin works in the body, it is important to understand the absorption and metabolic profiles of luteolin and its bioavailability in vivo. Although there are still few studies^9,10^, luteolin is presumed to undergo intestinal absorption metabolism following oral intake.

Shimoi et al. reported the absorption and metabolism of luteolin to its glucuronide conjugates following its oral administration in rats^9^. As the conjugation sites of the luteolin glucuronides had not been determined, we recently investigated the position of the glucuronide group in these metabolites and confirmed the presence of luteolin-3'-O-β-D-glucuronide (luteolin-3'G), luteolin-4'-O-β-D-glucuronide (luteolin-4'G), and luteolin-7-O-β-D-glucuronide (luteolin-7G) (Fig. 1) in rat and human plasma following the oral administration of luteolin, with luteolin-3'G as the major metabolite^11,12^. Interestingly, these metabolites also exhibited anti-inflammatory activities in vitro, though they were not as potent as luteolin^12^. Based on this evidence, it is likely that not only luteolin but also its metabolites (e.g., luteolin-3'G, luteolin-4'G, luteolin-7G) are responsible for the reported effects of luteolin in vivo.

On another note, flavonoids, including luteolin, possess various potential benefits, however, their low bioavailability^13^ often hinder their optimal activities in vivo. To overcome this problem, recent studies have applied microemulsion system to improve the delivery of poorly water-soluble flavonoids and polyphenols (e.g., quercetin^14^ and curcumin^15^). In case of luteolin, to the best of our knowledge, there has only been one report regarding the administration of luteolin microemulsion (LT-ME) to rats^16^. While this study showed an increase in the plasma AUC level following LT-ME intake, it is unclear whether the luteolin metabolites are included in the measurement of plasma luteolin^16^. Considering the physiological role of luteolin metabolites^12^, in order to assess the extent to which the oral bioavailability of luteolin is actually improved by microemulsion system, it is essential to understand the metabolic profile of luteolin.

Based on the above findings, the present study aimed to evaluate the absorption and metabolic profile of luteolin delivered in microemulsion system via oral intake. For these purposes, we first prepared water-dispersed luteolin (WD-L) using the microemulsion system and evaluated their dispersibility in water by particle size distribution. Next, we compared the absorption and metabolic profiles of free luteolin (CO-L) with WD-L. As a result, we found that WD-L increases the oral bioavailability of luteolin (i.e., increased plasma levels of luteolin-3'G, luteolin major metabolite) without affecting the metabolic profile. This evidence thereby provides a solid basis for future application of the microemulsion-based approach (i.e., WD-L) to enhance the bioavailability of luteolin in food products or supplements.

## Result and discussion

### The necessity of evaluating the absorption and metabolic profile of luteolin following oral administration of WD-L

Luteolin possesses many functional properties, such as anti-inflammatory, antioxidant activities, and anticancer properties^4–6^. Despite its various beneficial effects, luteolin, just like other types of flavonoids, has low bioavailability^13^. In attempts to improve the oral in vivo bioavailability of flavonoids and polyphenols, previous studies have suggested the use of a microemulsion-based delivery system^14–16^. In regards to luteolin, only one study reported the absorption profile of luteolin delivered in microemulsion system ^16^. However, it is not specified whether the luteolin metabolites are included in the measurement of plasma luteolin and thus, it is unclear the extent to which this delivery method can improve the bioavailability of luteolin. Therefore, our present study was aimed to develop WD-L using microemulsion system and understand in vivo the bioavailability in rats following oral administration of WD-L.

### Preparation of WD-L and evaluation of its water dispersibility

**Figure 2:**
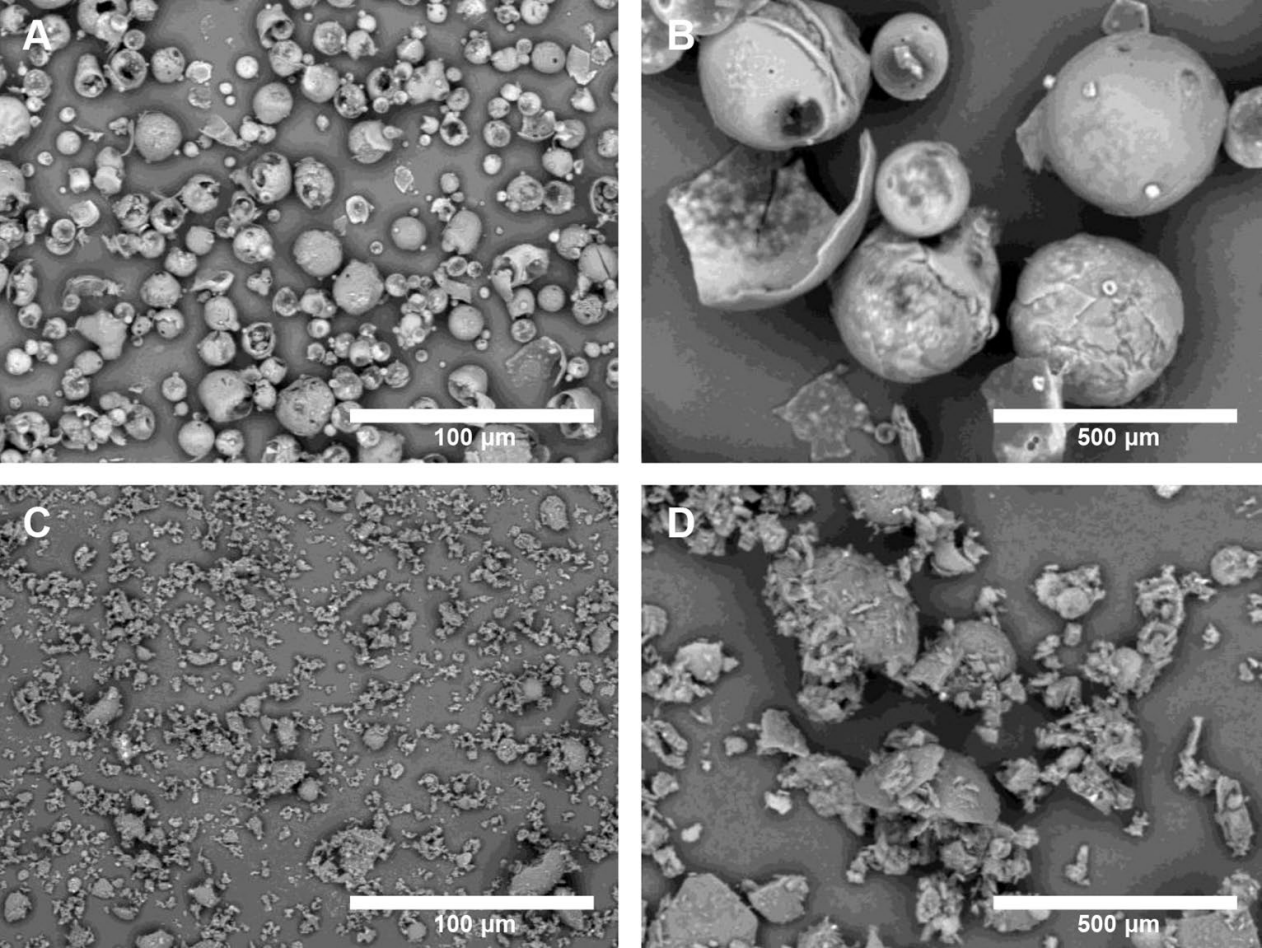
Surface morphology of WD-L powder (A: × 100, B: × 500 magnification) and CO-L powder (C: × 100, D: × 500 magnification), analyzed by SEM.

To prepare WD-L, we started by determining the optimal formulation using materials that are often used for producing microemulsion in the past studies, including: (1) saponin, a surfactant containing hydrophobic triterpenoids and hydrophilic sugar that is commonly used to stabilize the emulsion and prevent aggregation of hydrophobic substances; (2) dextrin (a type of starch) as excipient; and (3) gum Arabic, a highly polymerized polysaccharide that is commonly used as a thickener in food products or supplements. Thus, in the present study, we tried different variations of conditions and determined the ideal ratio of saponin, gum Arabic, and dextrin that can yield WD-L with good physicochemical properties^17^. The above components were mixed with luteolin (i.e., CO-L) and emulsified, then underwent spray-drying to yield WD-L containing 19% luteolin. The scanning electron microscope (SEM) analysis of the samples showed that WD-L’s particles are made up of thin-shelled sphere structures with small variations in size, whereas CO-L’s particles are irregular in shape and vary in size (Fig. 2).

**Figure 3:**
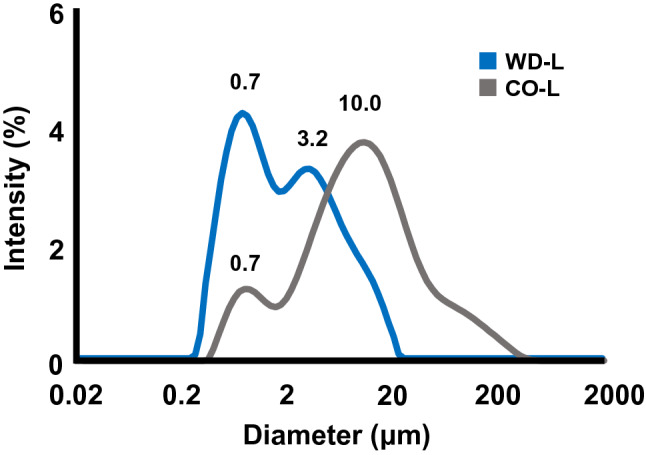
Particle size distributions of WD-L and CO-L.

The size of particles can be an indicator of their dispersibility in water. The cohesive force of the powder agglomerates is generally lower in the dispersed state; thus they present mainly as single particles. Therefore, we evaluated the dispersibility of WD-L and CO-L by measuring their particle size distribution (Fig. 3, Table 1). Our data shows that CO-L had a broad particle size distribution from D_10_ = 1.1 µm to D_90_ = 48.6 µm, with the most frequent diameter and volume mean diameter of 10.0 µm and 20.1 µm, respectively. Meanwhile, WD-L had a narrower distribution from D_10_ = 0.5 µm to D_90_ = 7.7 µm, with the most frequent diameter and volume mean diameter of 0.7 µm and 3.0 µm, respectively. The results of the particle size distribution analysis suggested that the peak width of CO-L is broader than that of WD-L (which also coincide with their polydispersity index (PDI), as seen in Table 1). The main peak (the most frequent diameter) of WD-L overlapped with the side peak (the second most frequent diameter) of CO-L at 0.7 µm, and no smaller peaks were found, thus indicating that 0.7 µm is the size of a single particle and WD-L is most likely to exist mainly as single particles in water. As for the aggregates, the side peak of WD-L (3.2 µm) was smaller than the main peak of CO-L (10 µm), suggesting a decrease in the cohesion of WD-L in water. Considering the results of the SEM analysis, thin-shelled sphere structures of WD-L may have disintegrated in the water and dispersed as fine particles. Furthermore, when CO-L and WD-L were dispersed in water and left to stand after 5 min, CO-L immediately precipitated whereas WD-L remained dispersed. These results suggest that the dispersibility of WD-L is superior to that of CO-L, as it exists in water mostly as single particles rather than aggregates. To understand the factors contributing to the good dispersibility of WD-L, we measured the absolute value of zeta potential. The results showed a smaller value for WD-L compared to CO-L (Table 1), and thus, it is likely that the good dispersibility of WD-L is due to its particle size and emulsibility rather than inter-particle attraction. As a side note, since the above experiments were conducted in water (neutral condition), it would be interesting to investigate the water dispersibility of WD-L under various conditions (e.g., at low pH conditions, where proteins tend to aggregate) in the future. Then, in the next experiment, we investigated whether the delivery of luteolin in a microemulsion system improves its in vivo bioavailability in rats.

**Table 1:** Detailed information on particle characterization of WD-L or CO-L.

	WD-L	CO-L
D_10_ (µm)	0.5	1.1
D_50_ (Median diameter) (µm)	1.6	8.4
D_90_ (µm)	7.7	48.6
Most frequent diameter (µm)	0.7	10.0
D [4, 3] (volume mean diameter) (µm)	3.0	20.1
PDI	0.37	0.74
Zeta potential (mV)	− 30.6	− 45.0

PDI, polydispersity index.

### Characterization of absorption and metabolic profile of luteolin following WD-L intake

#### Determination of sample dose and blood collection time

In the previous studies^11,12^, we developed an LC–MS/MS method to detect and measure the concentration of luteolin and its metabolites in rat and human plasma. Using this method, we investigated the absorption and metabolism of luteolin within 24 h after its oral administration (20 mg/kg B.W.) in rats. We found that the main luteolin’s metabolite, luteolin-3'G, reached the maximum level within 3 h of luteolin intake and remained detectable up to 12 h. In the present study, we determined the dosage and blood collection based on the conditions used in our previous studies (as mentioned above^11,12^). Thus, we administered WD-L and CO-L (equivalent to 20 mg/kg B.W. luteolin) to rats and collected blood samples within 24 h following oral administration. We then analyzed the plasma concentrations of luteolin and its metabolites using LC–MS/MS.

#### Metabolic profile of WD-L and CO-L intake in rat plasma

**Figure 4:**
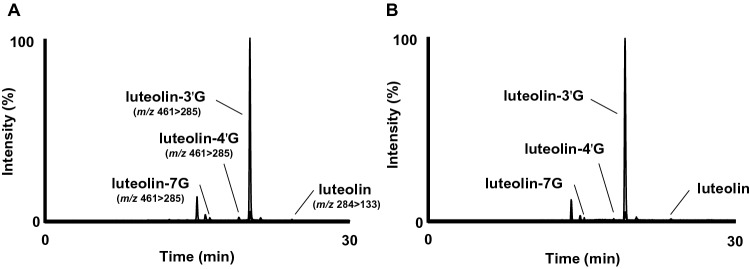
LC–MS/MS chromatograms of luteolin-3'G, luteolin-4'G, luteolin-7G, and luteolin in rat plasma at 3 h after oral administration of WD-L (**A**) and CO-L (**B**).

We did not detect any peaks corresponding to luteolin or its metabolites in rat plasma before oral administration of CO-L and WD-L. Within 3 h after the administration of both samples, we detected the highest peak that corresponded to luteolin-3'G with some smaller peaks that correspond to intact luteolin, luteolin-4'G, and luteolin-7G in rat plasma (Fig. 4). From these results, luteolin-3'G presents as the major luteolin metabolites in WD-L, as well as CO-L. Plasma levels of luteolin-3'G reached a maximum concentration at 3 h in both groups and remained at detectable levels up to 12 h. It then reached trace level or no longer detected after 24 h. These results were in line with our previous findings ^11,12^. More importantly, we found that the metabolic profile of luteolin was similar for both CO-L and WD-L. Thus, it is clear our microemulsion system in WD-L has little or no effect on the metabolism of luteolin. This also indicates that luteolin’s glucuronidation by the phase II metabolic enzymes during absorption and after absorption are unaffected in microemulsion system^7^. As reference, we found that luteolin mainly presents as luteolin-3'G in plasma with only small portion of intact luteolin remains in plasma. Despite the abundance of luteolin-3'G in plasma, we previously found that its anti-inflammatory activity is actually less potent compared to intact luteolin in vitro^11^. Hence, the future study should verify the extent to which luteolin-3'G (high abundance/low activity) and/or intact luteolin (low abundance/high activity) can contribute to the reported physiological functions of luteolin in vivo. Overall, this paragraph shows that the microemulsion process barely changed the metabolic profile of luteolin. To further investigate the absorption amount of luteolin in the body, we quantified plasma concentration of luteolin and luteolin metabolites during administration CO-L and WD-L.

#### Improvement of luteolin-3'G (luteolin-3'-O-glucuronide) in rat plasma

**Figure 5:**
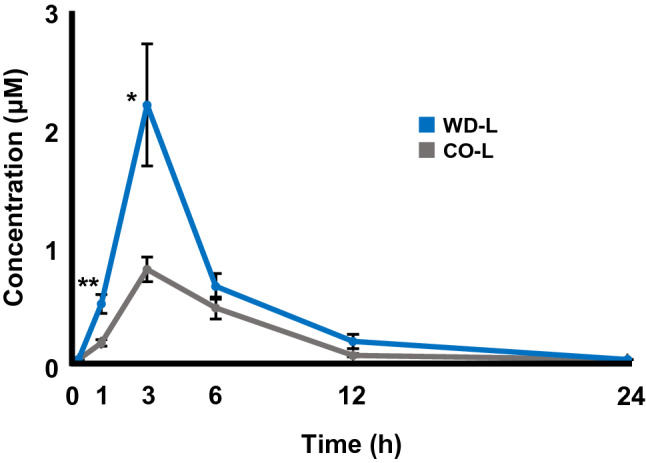
Plasma concentration of luteolin-3’G after administration of WD-L or CO-L (equivalent to 20 mg luteolin/kg body weight). Data was shown as mean ± SE (*n* = 8). *p < 0.05, **p < 0.01 vs. CO-L.

In this section, we investigated the amount of luteolin and its metabolites in plasma following oral administration of CO-L or WD-L. Using the standard calibration curve of luteolin-3'G, we were able to determine the time-dependent change in plasma luteolin-3'G level at 0, 1, 3, 6, 12, and 24 h after administration of WD-L or CO-L (Fig. 5) and calculated the area under the plasma concentration–time curve (AUC) (Table 2). Plasma concentration of luteolin-3'G, the main metabolite of luteolin, reached the maximum level at 3 h in both CO-L and WD-L groups, suggesting that luteolin was absorbed at a similar rate in both groups. We discovered that WD-L group has higher plasma levels of luteolin-3'G than the CO-L group at any given time points. In particular, the differences were statistically significant at 1 h and 3 h time points, leading to a significant increase in the AUC (2.2-fold increase) following the administration of WD-L group compared to the CO-L group, thus indicating higher bioavailability of luteolin in WD-L.

**Table 2:** The AUCs of luteolin-3’G after administration WD-L or CO-L (equivalent to 20 mg luteolin/kg body weight).

	WD-L	CO-L
AUC (µM/h)	8.42 ± 1.06**	3.85 ± 0.52

Data were shown as mean ± SE (*n* = 8). **p < 0.01 vs. CO-L.

To understand the mechanisms that contribute to the improvement in bioavailability following WD-L intake, studies on curcumin, a polyphenol with similar polarity to luteolin, may be of reference. A previous in vitro study reported that the saponin-based nanoparticle system increases the bioaccessibility of curcumin by 6.9-fold^18^. Besides, this study reported that rats given the curcumin nanoparticles also show a tenfold increase in the AUC of plasma curcumin level compared to rats given the free curcumin. In this study, saponin in the curcumin nanoparticle would be responsible in promoting the membrane permeability of curcumin and its passing through the intercellular tight junctions that exist in the interstices between gastrointestinal epithelial cells^18,19^. As both this study and our present study on WD-L used saponin in the delivery system, it is possible that similar mechanism underlie the improvement in luteolin’s bioavailability in microemulsion system.

On another note, food compounds need to pass through the unstirred water layer before reaching the surface of the gastrointestinal membrane; this is probably the reason for lower intestinal absorption of water-insoluble food compounds such as luteolin. Our result in the previous section showed that WD-L has higher water dispersibility. Thus, it is likely that WD-L pass through the unstirred water layer with more ease, resulting in higher absorption of the ingested luteolin through the intestinal lumen.

Another factor that may account for the increased permeability of water-insoluble compounds is their particle sizes. For instance, despite not using microemulsion system for delivering luteolin, a study by Lu et al. attributed the smaller luteolin particle size (53 nm) to the 2.1-fold increase in luteolin absorption^20^. In the present study, our microemulsion system yielded WD-L with a particle size of 1.6 µm. In the future studies, we would like to verify whether this particular size provides any advantage for the intestinal absorption of luteolin. In addition, studies have shown that the antioxidant activity of luteolin increases as its particle size was made smaller (e.g., from 22.7 μm to 2.3 μm in Santos, A. et al.^21^, from about 2 mm to 15.6 μm in Speroni, C. et al.^22^); thus, it will also be interesting to see whether WD-L has better antioxidant activity than CO-L.

In conclusion, saponin-based luteolin microemulsion showed higher water dispersibility (probably due to its smaller particle sizes), which contributes to its higher oral bioavailability. As mentioned in the Introduction section, to the best of our knowledge, only one study reported an increase in luteolin absorption following intake of luteolin using microemulsion system^16^. While both this study and our present study reported the increase in plasma AUC of luteolin when delivered in microemulsion system, due to the differences in the processing conditions used (i.e., emulsion composition ratios, types of surfactants, and emulsion creation equipment), it is perhaps difficult to draw a direct comparison between these studies. Therefore, future studies should verify how different microemulsion composition affect the improvement in luteolin’s oral bioavailability and further elucidate how WD-L improves the bioavailability of luteolin. Moreover, because we previously reported that the metabolic profiles of luteolin are partially different between human and rat^12^, in the future, we would also like to investigate the effect of WD-L on luteolin metabolism and whether WD-L remains as effective in human.

## Material and method

### Chemicals

Luteolin standard for liquid chromatography-tandem mass spectrometer (LC–MS/MS) analysis was purchased from Tokyo Chemical Industry Co., Ltd. (Tokyo, Japan). Luteolin-3'G, luteolin-4'G, and luteolin-7G were prepared according to our previous study^11^. A Chrysanthemum (*Chrysanthemum morifolium Ramat*) flower extract (50% luteolin) used to prepare WD-L was obtained from Hangzhou Skyherb Technologies Co., Ltd. (Zhejiang Province, China). Quillaja saponin was purchased from Maruzen Pharmaceuticals Co., Ltd. (Hiroshima, Japan). Dextrin was obtained from San-ei Sucrochemical Co., Ltd. (Aichi, Japan). Gum Arabic was obtained from Nippon Funmatsu Yakuhin Co., Ltd. (Osaka, Japan).

### Preparation of water-dispersed luteolin (WD-L)

First, Quillaja saponin (20 g) was mixed with pure water (300 mL) and heated to 80 °C to stabilize the emulsion. Into the solution, luteolin (100 g), dextrin (100 g) as an excipient, and gum Arabic (30 g) as a thickener were then added and dissolved to maintain the stability of the emulsion. The mixture was then emulsified at 500 kg/cm^2^ for 3 min for 3 times using a high-pressure homogenizer (ECONIZER LABO-01; Sanmaru Machinery Co., Ltd) and spray-dried (Mini Spray Dryer GB22; Yamato Scientific) to yield WD-L powder^17^.

### Particle characterization of WD-L and CO-L

The surface morphology of WD-L powder and CO-L powder were evaluated by SEM (TM4000 Type II; Hitachi Ltd, Tokyo, Japan). The particle size of WD-L powder and CO-L powder were analyzed using the Laser Diffraction Particle Size Distribution Analyzer LMS-2000e (Seishin Enterprise Co., Ltd.) in the range from 0.2 µm to 2000 µm as volume diameter. WD-L powder and CO-L powder were dispersed in water and mixed by sonication before the analysis. The average particle size for each sample was presented as volume mean diameter. The PDI and zeta potentials of WD-L powder and CO-L powder were analyzed by dynamic light scattering and laser doppler anemometry using ELS-Z (Otsuka Electronics Co., Ltd, Osaka, Japan).

### Animal study

Male Sprague–Dawley rats (8 week-old) were obtained from CLEA Japan, Inc. (Tokyo, Japan) and housed in cages maintained at 23 °C with a 12 h light/dark cycle. The rats were acclimatized with free access to water and commercial rodent chow (CE-2; CLEA Japan Inc.) for one week. After the acclimatization period, we performed a cross-over experiment after 12 h of fasting. We administered either a single oral dose of WD-L or CO-L to rats at an intake dose equivalent to 20 mg/kg B.W. At 0, 1, 3, 6, 12, and 24 h after sample administration, blood was collected from the tail vein using a capillary tube and centrifuged (1000 × *g*, 15 min, 4 °C) to obtain the plasma. After one week of washout period, we then gave each group the alternate treatment. All animal experiments were conducted based on the ARRIVE guidelines and the Animal Experiment Guidelines of the Institutes for Animal Experimentation at Tohoku University. The protocol for animal experiments was approved by the Center for Laboratory Animal Research, Tohoku University (Approval number: 2020-AgA-022).

### Extraction of luteolin and its metabolites from rat plasma

First, rat plasma (100 μL) was mixed with acetonitrile (300 μL) and centrifuged (1000 × *g*, 10 min, 4 °C). Then, the supernatant was collected (first batch), while the precipitate was mixed with methanol (300 μL) and centrifuged (1000 × *g*, 10 min, 4 °C). The supernatant was collected and combined with the first batch of supernatant, then dried using a centrifugal evaporator and redissolved in a 10% acetonitrile aqueous solution.

### Detection and quantification of luteolin and its metabolites using a high-performance liquid chromatography-tandem mass spectrometry (HPLC–MS/MS) system

To determine the plasma level of luteolin and its metabolites, a 10 μL sample of the extract was subjected to an HPLC–MS/MS system consisting of a liquid chromatography system (Agilent, Tokyo, Japan) and a 4000 QTRAP HPLC–MS/MS (SCIEX, Tokyo, Japan), with analytical parameters that were similar to our previous study^11,12^. Chromatographic separation of luteolin and its metabolites were done on a C18 column (CAPCELLPAK C18 MGII S3, 4.6 × 150 mm; Shiseido, Tokyo, Japan) at a flow rate of 0.8 mL/min and temperature maintained at 40 °C. Gradient elution was performed using water containing 0.1% trifluoroacetic acid (mobile phase A) and acetonitrile (mobile phase B). The gradient profile was as follows: 0–20 min, 10–30% B linear; 20–25 min, 30–50% B linear. The mobile phase was split so that the eluate entered the HPLC–MS/MS system at a flow rate of 0.2 mL/min. Luteolin, luteolin-3'G, luteolin-4'G, and luteolin-7G were analyzed in negative ion mode and detected by multiple reaction monitoring (MRM) for the transition of precursor ions to productions: luteolin (*m/z* 284 > 133), luteolin-3'G (*m/z* 461 > 285), luteolin-4'G (*m/z* 461 > 285), and luteolin-7G (*m/z* 461 > 285).

### Statistical analysis

Data are presented as mean ± standard error (SE). Unpaired two-tailed Student’s t-test was performed to assess the differences between the WD-L and CO-L groups. *P* values less than 0.05 were considered to be statistically significant.

## Data Availability

The datasets used and/or analyzed during the current study available from the corresponding author on reasonable request.

## References

[1] Cárdenas-Castro AP, Rochín-Medina JJ, Ramírez K, Tovar J, Sáyago-Ayerdi SG (2022). In vitro intestinal bioaccessibility and colonic biotransformation of polyphenols from mini bell peppers (*Capsicum annuum* L.). Plant Foods Hum. Nutr..

[2] Jeon IH (2014). Anti-inflammatory and antipruritic effects of luteolin from perilla (*P. frutescens* L.) leaves. Molecules.

[3] Lin LZ, Lu S, Harnly JM (2007). Detection and quantification of glycosylated flavonoid malonates in celery, chinese celery, and celery seed by LC-DAD-ESI/MS. J. Agric. Food Chem..

[4] Harris GK, Qian Y, Leonard SS, Sbarra DC, Shi X (2006). Luteolin and chrysin differentially inhibit cyclooxygenase-2 expression and scavenge reactive oxygen species but similarly inhibit prostaglandin-E 2 formation in RAW 264.7 cells. J. Nutr..

[5] Igile GO (1994). Flavonoids from Vernonia amygdalina and Their Antioxidant Activities. J. Agric. Food Chem..

[6] Yan J (2012). Luteolin enhances TNF-related apoptosis-inducing ligand’s anticancer activity in a lung cancer xenograft mouse model. Biochem. Biophys. Res. Commun..

[7] Liang KL, Yu SJ, Huang WC, Yen HR (2020). Luteolin attenuates allergic nasal inflammation via inhibition of interleukin-4 in an allergic rhinitis mouse model and peripheral blood from human subjects with allergic rhinitis. Front. Pharmacol..

[8] Kempuraj D (2021). Neuroprotective effects of flavone luteolin in neuroinflammation and neurotrauma. BioFactors.

[9] Shimoi K (1998). Intestinal absorption of luteolin and luteolin 7-O-β-glucoside in rats and humans. FEBS Lett..

[10] Agrawal, P. K. Natural Product Communications: Editorial. *Nat. Prod. Commun.* **9**, (2014).

[11] Kure A (2016). Metabolic fate of luteolin in rats: Its relationship to anti-inflammatory effect. J. Agric. Food Chem..

[12] Hayasaka N (2018). Absorption and metabolism of luteolin in rats and humans in relation to in vitro anti-inflammatory effects. J. Agric. Food Chem..

[13] Thilakarathna SH, Vasantha Rupasinghe HP (2013). Flavonoid bioavailability and attempts for bioavailability enhancement. Nutrients.

[14] Tang TT, Hu XB, Liao DH, Liu XY, Xiang DX (2013). Mechanisms of microemulsion enhancing the oral bioavailability of puerarin: Comparison between oil-in-water and water-in-oil microemulsions using the single-pass intestinal perfusion method and a chylomicron flow blocking approach. Int. J. Nanomedicine.

[15] Jain S, Jain AK, Pohekar M, Thanki K (2013). Novel self-emulsifying formulation of quercetin for improved in vivo antioxidant potential: Implications for drug-induced cardiotoxicity and nephrotoxicity. Free Radic. Biol. Med..

[16] Liu Y (2014). Nanostructured lipid carriers versus microemulsions for delivery of the poorly water-soluble drug luteolin. Int. J. Pharm..

[17] Takahashi, H. et al. Luteolin-containing composition and method for manufacturing same. (Japan/Shizuoka Patent No. W O 2019/070056 A 1) (2019)

[18] Peng S (2018). Improving curcumin solubility and bioavailability by encapsulation in saponin-coated curcumin nanoparticles prepared using a simple pH-driven loading method. Food Funct..

[19] Moghimipour E, Tabassi SAS, Ramezani M, Handali S, Lobenberg R (2016). Brush border membrane vesicle and Caco-2 cell line: Two experimental models for evaluation of absorption enhancing effects of saponins, bile salts, and some synthetic surfactants. J. Adv. Pharm. Technol. Res..

[20] Wang L (2019). Preparation and characterization of luteolin nanoparticles for enhance bioavailability and inhibit liver microsomal peroxidation in rats. J. Funct. Foods.

[21] Santos A (2022). Micronization of luteolin using supercritical carbon dioxide: Characterization of particles and biological activity in vitro. J. Supercrit Fluids.

[22] Speroni C (2021). Micronization increases the bioaccessibility of polyphenols from granulometrically separated olive pomace fractions. J. Funct. Foods.

